# Metagenomic Approaches to Investigate the Contribution of the Vineyard Environment to the Quality of Wine Fermentation: Potentials and Difficulties

**DOI:** 10.3389/fmicb.2018.00991

**Published:** 2018-05-16

**Authors:** Irene Stefanini, Duccio Cavalieri

**Affiliations:** ^1^Division of Biomedical Sciences, University of Warwick, Coventry, United Kingdom; ^2^Department of Biology, University of Florence, Florence, Italy

**Keywords:** wine, metagenomics, bacteria, fungi, vineyard, environment

## Abstract

The winemaking is a complex process that begins in the vineyard and ends at consumption moment. Recent reports have shown the relevance of microbial populations in the definition of the regional organoleptic and sensory characteristics of a wine. Metagenomic approaches, allowing the exhaustive identification of microorganisms present in complex samples, have recently played a fundamental role in the dissection of the contribution of the vineyard environment to wine fermentation. Systematic approaches have explored the impact of agronomical techniques, vineyard topologies, and climatic changes on bacterial and fungal populations found in the vineyard and in fermentations, also trying to predict or extrapolate the effects on the sensorial characteristics of the resulting wine. This review is aimed at highlighting the major technical and experimental challenges in dissecting the contribution of the vineyard and native environments microbiota to the wine fermentation process, and how metagenomic approaches can help in understanding microbial fluxes and selections across the environments and specimens related to wine fermentation.

## Introduction

Wines made from identical vine cultivars and under the same conditions can be recognized for their distinctive features encompassing chemical composition (Son et al., 2009; Perestrelo et al., 2014; Ziółkowska et al., 2016) and sensory characteristics (Van Leeuwen and Seguin, 2006; Van Leeuwen, 2010; Robinson et al., 2012; Hopfer et al., 2015) due to the different regional origins. The French word for “soil” (also “land”), *terroir*, was adopted to refer to the interaction between the plants, the environment and human factors (Gladstones, 2011) and nowadays it is frequently used to relate wine sensory attributes to its geographic origin (Van Leeuwen and Seguin, 2006). Recently, several studies have shown that the differences between grapes or fermenting musts from different regions are mirrored by geographic variation of the microbial community compositions (Bokulich et al., 2014; Gilbert et al., 2014; Taylor et al., 2014; Morrison-Whittle and Goddard, 2015; Pinto et al., 2015; Belda et al., 2017). In addition, the differences among microbial populations have been shown to be correlated with the organoleptic characteristics of fermenting musts (Knight et al., 2015) (Bokulich et al., 2016). The reasons why microbial communities differ among geographic locations are still far to be fully understood. However, recent studies shaded some light on this topic, highlighting that microbial populations found in musts may originate from the native environment surrounding the vineyard (Morrison-Whittle and Goddard, 2018) and that the geographical differences among populations were more evident for fungi than for bacteria (Miura et al., 2017). Because of the observation of a putative microbial terroir, the role and persistence of environmental microbial species in the wine fermentative process gained a renewed interest.

The compositions of microbial populations present in the vineyard, in the winery, and in fermenting musts have been extensively investigated by means of traditional microbiological methods (Morgan et al., 2017). Culture-based approaches relied on the isolation of microbes on laboratory media, and their identification and characterization through biochemical assays, microscopy, and molecular biology. Nevertheless, culture-based methods often failed to identify microorganisms present at low frequency in the sample and non-culturable cells. In 1999, Ampe and collaborators showed that at least 25–50% of the microbial community could not be cultured in laboratory conditions, hence clearly highlighting the drawbacks of culture-based approaches (Ampe et al., 1999). In addition, the use of only biochemical and phenotypic characteristics to identify microbes was shown to be inadequate, likely because parallelism and reversals of phenotypes occurred in species evolution (Kurtzman and Robnett, 1994; Guzmán et al., 2013). As an example, the *Candida* genus, initially intended to include all the “asexual yeasts that divide by multilateral budding but have no distinctive cellular morphology” (Daniel et al., 2014), is now recognized as a polyphyletic genus and undergoing revision to make species grouping consistent with phylogenetic affinities (Daniel et al., 2014). These limitations highlighted the need to develop culture-independent techniques enabling the rapid, accurate, and exhaustive description of microbial populations. Next Generation Sequencing (NGS) approaches fulfilled these needs, allowing the identification of both bacteria and fungi present in complex samples such as grapes, musts, and fermentations (Morgan et al., 2017).

## Metagenomic approaches

Thanks to the advent of NGS, several metagenomic approaches are nowadays available to dissect the composition of microbial populations. The available sequencing techniques have already been reviewed by Morgan et al. (2017), and this review is aimed at highlighting the potentials of different metagenomic approaches grouped as amplicon-based and whole-genome sequencing. The first group is based on the sequencing of target sequences known to be able to distinguish microbes, the latter group allows the sequencing of the complete pool of DNA extracted from a given sample.

### Amplicon-based metagenomic approaches

Amplicon-based approaches, also called metabarcoding, rely on the contemporary sequencing of the same DNA sequence shared by all the microbes present in a given sample, but different enough to allow the identification of different microorganisms (Table 1).

**Table 1:** Advantages and drawbacks of amplicon-based and whole-genomics sequencing approaches.

	**Advantages**	**Drawbacks**	**Organism**	**Region**	**Advantages**	**Drawbacks**
Amplicon-based sequencing	Large and comprehensive reference databases are available Several pipelines available for bioinformatics analysis Detection of rare taxa Taxonomy to the genus level (species at best)	Biased relative quantification of bacterial communities: bacterial species bear various number of copies of 16S rRNA genes Functional annotation can only be inferred Sequencing of matrix (e.g., grape ITS, chloroplast 16S) Low confidence for taxonomic assignment at the species level	Bacteria	V1-V2 V6 (*16S rRNA)*		Overestimate richness
				V3, V7 V7-V8 (16S rRNA)		Underestimate richness
				V4, V5-V6 V6-V7 (16S rRNA)	Provide estimates comparable to those obtained with the complete 16S rRNA gene sequence	
			Fungi	ITS1	Detects more OTUs than D2 region	
				ITS2	Detects more OTUs than D2 region	
				D1-D2		
				18S rRNA gene		
Whole-genome sequencing	All microbes detected at once Taxonomic assignment at the species or strain level Functional annotations can be carried out by gene enrichment	May need available reference genomes Relative organism abundances vary significantly depending on the protocols adopted for DNA extraction and sequencing Generally, not deep enough to detect taxa present at low frequency in complex communities Amplification of sequencing of the matrix (e.g., grape)	

Back in 1977, Woese and Fox proposed the use of ribosomal RNA (rRNA) sequences to determine the phylogenetic relations among organisms (Woese and Fox, 1977). rRNA sequences fulfilled the requirements for a good molecular marker: they are present in all the living organisms, their sequences present conserved regions suitable as targets for primers used in polymerase chain reactions (PCR) but differ enough between species to discriminate them (Woese and Fox, 1977). The pioneering proposal of Woese and Fox is still relevant, as the rRNA sequences are currently used for amplicon-based metagenomics analyses. The typical target for bacterial metabarcoding is the 16S rRNA gene (Liu et al., 2007), while three regions are usually targeted in fungi: the ITS1-5.8S rRNA-ITS2, the 26S rRNA gene, and a region of the 18S rRNA gene (Xu, 2016).

#### Choosing the target region

By sequencing specific genes (or regions) one can identify the microbes at the genus or even at the species level. However, there are some limits in using the complete gene/region sequences for metagenomic analyses. First of all, the average reads length of NGS ranges from 150 to 300 bp, far shorter than the length of the target genes/regions which are ~1,500 bp for the 16S rRNA gene (Liu et al., 2007), 400–900bp for the ITS1-5.8S rRNA-ITS2 region (Esteve-Zarzoso et al., 1999), and >1,300 bp for the 26S rRNA gene (Pinto et al., 2014). The use of the whole IT1-5.8S rRNA-ITS2 fungal region for metagenomic purposes has an additional problem: the length of this region is not conserved among fungi (i.e., 400 bp in *Metschnikowia pulcherrima*, 880 bp in *Saccharomyces cerevisiae*) (Esteve-Zarzoso et al., 1999), and a preferential amplification may occur for shorter fragments. Hence, for metagenomics purposes, shorter regions have been selected from the full length of the target genes.

Nine hypervariable regions of the 16S rRNA gene sequence (V1-V9) have been targeted for the assessment of bacterial diversity (Liu et al., 2007). Unfortunately, the choice of partial sequence regions can significantly affect the results because the 16S rRNA gene regions have different divergence (Table 1; Youssef et al., 2009). Recent *in silico* studies showed the V4-V6 regions as the most reliable for the phylogenetic study of new phyla (Yang et al., 2016) and the V4, V5-V6, and V6-V7 regions as the most suitable regions for metagenomic purposes because providing estimates comparable to those obtained with the complete 16S rRNA gene sequence (Youssef et al., 2009). The sequencing of the V1-V2 region and the V6 region overestimated the species richness, while the sequencing of the V3, V7, and V7-V8 regions underestimated the species richness (Youssef et al., 2009). However, experiments did not confirm the results obtained with *in silico* analyses: the sequencing of the V3-V4 and V5-V6 from the same samples showed poor overlap in the lists of identified bacteria (Campanaro et al., 2014).

As for bacterial metabarcoding, even for fungal amplicon-based metagenomics choosing the proper fragment to be sequenced is pivotal. Again, comparative analyses have been carried out to assess which region is the most suitable for fungal metabarcoding (Table 1). For instance, Pinto and colleagues showed that the taxonomies identified in the same samples by sequencing the ITS2 region and the D2 domain of the 26S rRNA gene were only partially shared and that the ITS2 region identified a higher number of taxa than the D2 region (Pinto et al., 2014). In addition, the ITS1 and ITS2 region performances were compared by means of *in silico* and experimental analyses, revealing that the two regions gave highly similar results, but the ITS1 region allowed the identification of a greater number of taxa (Blaalid et al., 2013; Bokulich and Mills, 2013).

It is worth to mention that another problem raises when using metabarcoding for the dissection of the composition of microbial populations present in grapes and musts. In fact, being *Vitis vinifera* (and hence grapes) a eukaryote, it also bears the ITS1-5.8S rRNA-ITS2 region and 26S rRNA gene. Similarly, *V. vinifera* chloroplasts, being originated from cyanobacteria (Gray, 1989), bear the 16S rRNA gene. This implies that reads belonging to the matrix (grape or must) will be amplified and sequenced in metabarcoding, thus reducing the coverage for the associated microbial population. Hence, a particular care should be adopted in the extraction of microbial DNA, reducing at the minimum the amount of DNA from the matrix.

#### Available reference databases

An additional factor influencing the choice of the target to be used for microbial metabarcoding should be the availability of an exhaustive and curated reference database of annotated sequences. In fact, the taxonomic assignment is carried out through the comparison (e.g., alignment) of the sequenced regions with a database of annotated sequences. In principle, public repositories of sequences (i.e., GenBank) could be used as a source for reference sequences. Nevertheless, these repositories also encompass sequences amalgamated into the pseudo-divisions “environmental samples” and “unclassified,” worthless for taxonomic assignment in metabarcoding (DeSantis et al., 2006).

Several curated 16S rRNA databases are available, among which the most frequently used are RDP, Greengenes, SILVA, and LTP (Santamaria et al., 2012). Such resources, in addition to offering a curated list of annotated 16S rRNA sequences, also show additional functionalities. For instance, the RDP reference database can be used with the standalone program RDP Classifier for phylogenetic classification, and with LibCompare for comparison of taxa abundances between samples (Wang et al., 2007). Similarly, SILVA (Pruesse et al., 2007), LTP (Yarza et al., 2008), and Greengenes (DeSantis et al., 2006) reference databases can be used with the standalone program ARB for phylogenetic classification (Ludwig et al., 2004). While RDP, LTP, and Greengenes databases include complete 16S rRNA bacterial gene sequence, the SILVA database encompasses aligned sequences of the small (16S/18S, SSU) and large (23S/28S, LSU) rRNA subunits for all three domains of life.

While several reference databases are available for 16S rRNA bacterial sequences, just a few databases are available for fungal metabarcoding: UNITE (User-friendly Nordic ITS Ectomycorrhiza Database) (Abarenkov et al., 2010), ITS2 Database (Ankenbrand et al., 2015), and ITSoneDB (Santamaria et al., 2017). The lack of a wider range of available databases and tools specific for fungal metabarcoding can be ascribed to the relatively recent interest in fungal metagenomics and to the lack of a consensus in the selection of the target used for metabarcoding. While UNITE encompasses entire ITS1-5.8S rRNA-ITS2 sequences (Abarenkov et al., 2010), the ITS2 database includes sequences of the ITS2 region (Ankenbrand et al., 2015), and the ITSoneDB includes sequences of the ITS1 region (Santamaria et al., 2017).

#### Analytic tools and pipelines

The great success of amplicon-based metagenomic approaches encouraged researchers with various backgrounds to approach a technique that strongly relies on bioinformatics. Despite the collaboration of an expert bioinformatician being highly recommended to choose the best procedures, overcome with eventual unexpected outcomes of the analysis, and interpret the data, nowadays the availability of pipelines allows non-specialized researchers to handle and analyze metagenomic data. Such pipelines have been built by combining pre-existing tools and allowing the user to rapidly proceed through the steps of data processing without i.e., incurring the data conversion to meet the requirements of the used tool.

Once sequenced, amplicons need to be handled in a consequential series of steps: *i*- trim bases that have been flagged as low-quality by the sequencing platform; *ii*- (in case of paired-end sequencing) match and stitch paired reads; *iii*- remove artifacts such as chimeras (merged sequences wrongly paired); *iv*- filter out contaminant sequences (i.e., non 16S sequences); *v*- identify the Operational Taxonomic Units (OTUs) in the samples (e.g., clustering the entire set of sequences and then selecting a representative sequence for each cluster); *vi*- assign taxonomic identities to the OTUs by comparing the sequences to these present in reference databases. Such a set of processes has been variously implemented in the most frequently used pipelines such as mothur (Schloss et al., 2009), MICCA (Albanese et al., 2015), QIIME and QIIME2 (Caporaso et al., 2010), BioMaS (Fosso et al., 2015), the RDP's Pyrosequencing Pipeline (Cole et al., 2009), CloVR (Angiuoli et al., 2011), and CloVR-ITS (White et al., 2013) (the latter designed for fungal populations analyses).

Thanks to metagenomic analyses it is possible to describe and compare the compositions of microbial populations in almost every kind of sample. A step forward consists of the understanding of how changes in the composition of microbial communities impact the population's biological functions. Under the assumption that a given microbial taxon is uniformly able to perform specific biological functions [i.e., *Bacteroides* spp. might be inferred to contain genes encoding glycoside hydrolase activity (Xu et al., 2007)], it is possible to predict the functional profile of a given population from the taxon composition obtained by means of metabarcoding. Some tools have been generated with this aim, i.e., Tax4Fun (Aßhauer et al., 2015), PICRUSt (phylogenetic investigation of communities by reconstruction of unobserved states; Langille et al., 2013), and PanFP (pangenome-based functional profiles; Jun et al., 2015). All these tools are designed to infer functional profiles for bacterial populations. PICRUSt is based on the use of the Greengenes reference database (DeSantis et al., 2006) and the functional composition of reference genomes described in IMG (Markowitz et al., 2012). Briefly, OTUs are identified according to their clustering with taxa of the Greengenes database, and the biological function profile of the sample is inferred by the combination of functions described for the reference genomes corresponding to the taxa identified in the sample. Thus, PICRUSt predictions depend on the topology of the tree and on the distance to the next sequenced organism, limiting the analysis to well-characterized phyla (Aßhauer et al., 2015). Even Tax4Fun relies on the taxa identification by means of clustering against a reference database (SILVA Pruesse et al., 2007), but the SILVA-based 16S rRNA profiles are converted into taxonomic profiles based on the prokaryotic organisms in the KEGG database (Kanehisa and Goto, 2000) and finally the functions are inferred. PanFP works similarly to Tax4Fun, but in addition to KEGG, allows the inclusion of other gene annotation databases, e.g., Gene Ontology (Ashburner et al., 2000), Pfam (Punta et al., 2012), and TIGRFAMs (Haft et al., 2003).

However, it must be considered that those softwares cannot cope with lateral gene transfer or gene gain and loss, which may affect the ability to predict biological functions from taxonomy based on a single gene (Table 1). A further drawback of using DNA-based metagenomic data to infer the biological functions potentially exploited by microbial populations is that the detected DNA may belong to dead organisms. A few studies on the dynamics of microbial populations in fermentations reported the disappearance of DNAs belonging to microbes reasonably dying during the process (Marzano et al., 2016; Stefanini et al., 2016), hence suggesting a rapid degradation of DNA in this chemically hostile environment. However, an approach based on RNA sequencing would give a direct report of the functions achievable by the viable microbial populations.

### Whole-genome sequencing

Another NGS approach used to study the composition of microbial communities is whole genome sequencing. Instead of sequencing target DNA regions allowing microbial identification, whole-genome sequencing consists in the sequencing of all the DNA extracted from a given sample. The obtained sequences can be handled in various ways to identify the organisms present in the samples or to obtain other information. Hence, the composition of both fungal and bacterial populations can be dissected with a single round of whole-genome sequencing (Table 1).

Despite being unaffected by the problems highlighted for amplicon-based approaches, whole-genome sequencing has disadvantages. Indeed, it has been shown that, differently from amplicon-based sequencing, the relative organism abundances inferred from whole-genome sequencing may significantly vary according to the protocols used for DNA extraction and sequencing (Table 1; Gomez-Alvarez et al., 2009). In addition, whole-genome sequencing usually does not allow the identification of organisms present at low frequency in the sample (Table 1; Shah et al., 2011). However, a few direct comparisons of amplicon-based and whole-genome sequencing techniques revealed that the two approaches identify highly similar microbial populations, with the whole-genome sequencing approach capturing a higher level of diversity (more phyla and genera; Poretsky et al., 2014).

The reads obtained by means of whole-genome sequencing can be used not only to identify the microorganisms present in the sample but also to compare the relative abundances of bacteria and fungi (Cao et al., 2017). The main advantage of whole-genome sequencing over amplicon-based sequencing is its potential to characterize microbes at the species or even strain level (Cao et al., 2017). This topic is detailed in section Future Challenges for Metagenomic Approaches: the Sub-Species Level. Furthermore, the whole-genome sequencing approach also allows the direct identification of genes having relevant functional roles, whose presence could be only inferred with the amplicon-based metagenomic approach (see the previous section for further details), and thus, it is not affected by lateral gene transfer or deletion. In addition, this approach potentially allows the identification of functions previously unknown in certain organisms, even if the organisms do not have their genomes sequenced.

Several tools have been generated to obtain the microbial taxonomy profile from whole-genome sequencing data, among which the most used are Kraken (Wood and Salzberg, 2014), MetaPhlAn2 (Truong et al., 2015), riboFrame (Ramazzotti et al., 2015), and CLARK (Ounit et al., 2015). Other tools are available (e.g., TETRA, CompostBin, MEGAN, GRAMMY) and have been previously reviewed in Alaimo et al. (2018). Kraken and CLARK identify the percentages of reads aligning against a set of references genomes. riboFrame identifies reads overlapping the 16S rDNA genes through Hidden Markov Models and carries out the taxonomic assignment thanks to a naïve Bayesian classification. Hence, all reads identified as ribosomal are coherently positioned in the 16S rDNA gene, allowing the use of the topology of the gene to guide the abundance analysis. MetaPhlAn2 allows the species-level and strain-level profiling of bacteria, eukaryotes, and viruses, by means of sequence matching against a set of unique clade-specific marker genes identified from reference genomes (Table 1).

## Characterizing and comparing populations

In metagenomic analyses, populations are generally compared among samples having defined and known differences (i.e., the stage of grape maturation or the stage of must fermentation). Aiming to this, several measures are available to describe and compare the structure and composition of populations measuring the alpha biodiversity (within sample diversity) and the beta biodiversity (between samples diversity).

**Figure 1:**
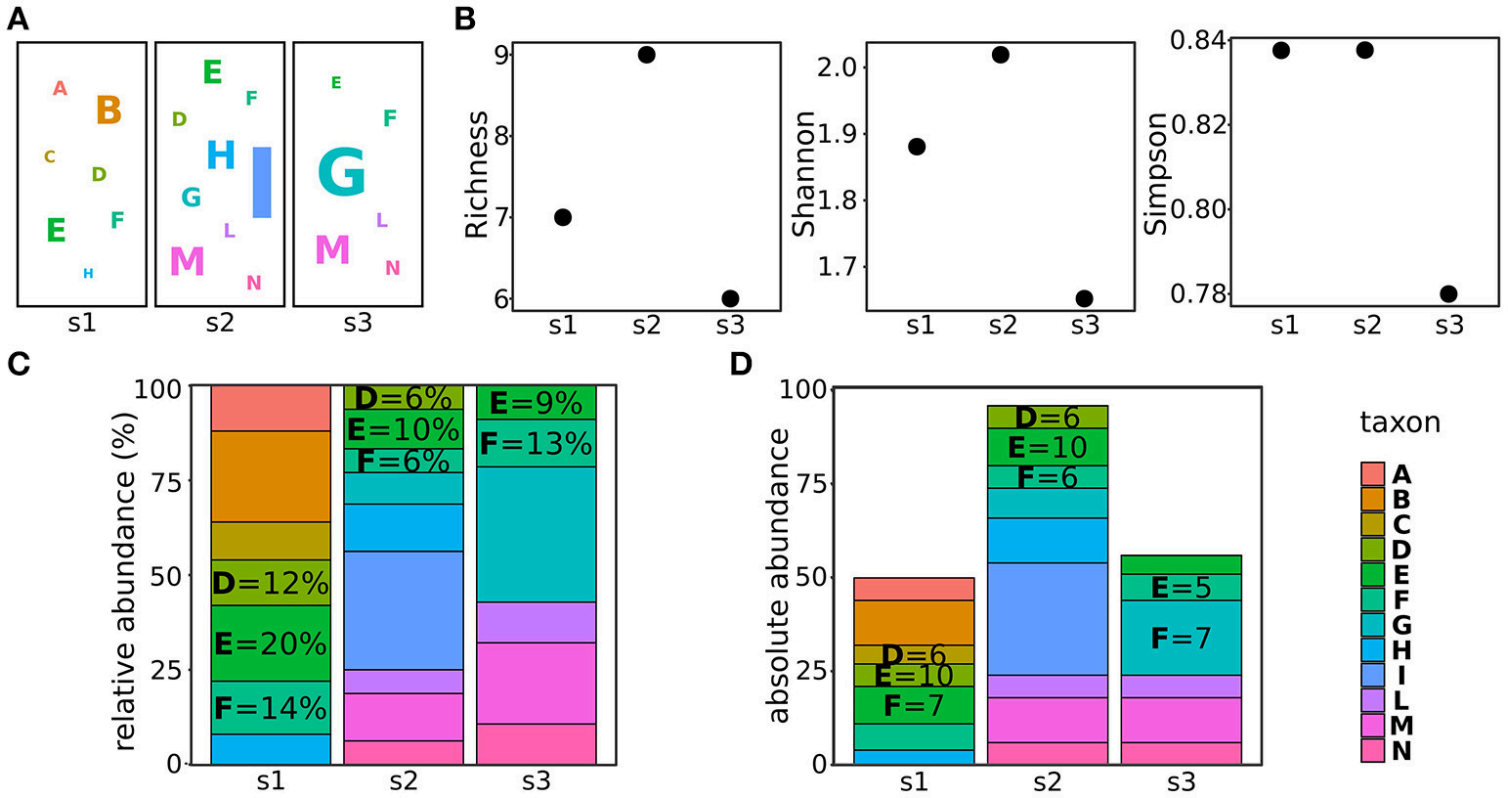
Characterizing and comparing populations. **(A)** Examples of three populations, letters refer to different taxa, the letter size indicating the abundance of the taxon in the sample. **(B)** alpha diversities (richness, Shannon index, and Simpson index) calculated for the populations shown in **(A)**. **(C)** taxa relative abundances of the taxa composing the populations shown in **(A)**. **(D)** taxa absolute abundances of the taxa composing the populations shown in panel **(A)**.

In metagenomics, three estimators are generally used to estimate the alpha biodiversity: richness, Simpson index, and Shannon index. The taxa richness is the number of different taxa present in the population, not considering their abundances. For example, the richness of the populations shown in Figure 1A is the number of different taxa (letters in the figure) present in the three populations (Figure 1B). The Simpson index is a measure of the population evenness, indicating the probability that two randomly sampled individuals belong to two different taxa (i.e., species) (Lugtenberg and Kamilova, 2009). Hence, it considers both the richness and the abundances of the identified taxa: the more equal the proportions for each of the taxa, the more homogeneous, or even, they are (Simpson, 1949). As the Simpson index, the Shannon index combines both evenness and richness, but it quantifies the uncertainty in the taxon identity of a randomly chosen individual (Tuomisto, 2012). In plain terms, if the population is composed by many taxa present at the same frequency, all the randomly chosen individuals will have the same (low) probability of being assigned to the correct taxon, hence, the uncertainty (Shannon index) will be high. On the contrary, if a large part of the population belongs to a given taxon, the probability of correctly assigning the randomly chosen individual will be high, thus reducing the Shannon index (Shannon, 1948). The major difference between the Shannon and the Simpson indexes is that the first gives a higher weight to rare taxa. Hence, the population with a low richness (s1 in Figure 1A) will have a lower Shannon index compared to a population with a higher richness (s2 in Figure 1A) if the first population encompasses more rare taxa than the second (Figure 1B). On the contrary, the Simpson index of the first population will be comparable or higher than the Simpson index of the second population (Figure 1B).

Two beta diversities are usually used in metagenomic analyses: the Bray-Curtis dissimilarity and the UniFrac distance. The Bray-Curtis dissimilarity is a measure of the differences in composition between two samples based on taxa abundances in each sample (Tuomisto, 2012). The UniFrac distance, devised by the Knight group at the University of Colorado, incorporates the phylogenetic distances between taxa, and can include the information on the abundance of taxa (weighted UniFrac) or simply consider the presence/absence of taxa (unweighted UniFrac; Borcard et al., 2011).

When delving into the details of the components of the microbial population, it is worth to make a consideration of abundances. Usually, the abundances of taxa are reported as relative abundances (the percentage of counts of the given taxon on the total of counts in the sample) (Figure 1C). This measure is fairly used to indicate how the proportion of taxa changes in different samples. However, when comparing bacterial populations, it must be considered that the 16S copy number varies greatly among different bacteria (Lozupone et al., 2007; Kembel et al., 2012) and this obviously affects the quantification of bacterial abundances in different samples (Větrovský and Baldrian, 2013). To cope with this problem and properly compare the abundances of bacteria, tools such as CopyRighter (Angly et al., 2014) and rrnDB (Klappenbach et al., 2001) have been created to scale the abundances according to the known number of 16S copies in different bacteria.

In addition, a further care should be used especially when analyzing dynamic processes such as must fermentations. The amount of microbes present in grapes before harvesting, is known to exponentially increase during the late phases of grape maturation (from 10^2^-10^4^ cells per grape before maturation to 10^7^-10^9^ per grape in damaged, ripen grapes; Mortimer and Polsinelli, 1999; Kembel et al., 2012) and even more during the early phases of fermentation, when free sugars are available and microbes find a more suitable environment (Mortimer and Polsinelli, 1999; Barata et al., 2012). On the other hand, the increasing amount of ethanol produced by fermenting yeasts progressively selects the most sensitive species, reducing the biodiversity of the sample and potentially modifying the total amount of present microbes (see further details in section Metagenomic From Vineyard to Wine; Goddard, 2008). Because of these fluctuations of the size of microbial populations, the use of proportions to compare the abundances of taxa in different samples might not be suitable. For instance, the same amount of a taxon in populations of different sizes (i.e., taxon E in samples s1 and s2, Figure 1D) will result in different relative abundances (20 and 10% in sample s1 and s2, respectively). Hence, it is not possible to obtain information on the individual fitness (or persistence) of taxa during the process from relative abundances. To help in this comparison, we recently applied an approach allowing us to scale the relative abundances, obtained through amplicon-based metagenomics, according to the total amount of microbes identified in the sample (Stefanini et al., 2016). This approach, based on the quantification of the total amount of fungal or bacterial DNA in a given sample through quantitative real-time PCR (qRT-PCR) allowed us to gain insightful information on the evolution of microbial populations before and during the fermentation process (Stefanini et al., 2016).

## Metagenomic from vineyard to wine

Despite wine fermentation is usually associated to the process of sugar conversion into ethanol, the production of wine is nowadays known to be influenced also by the characteristics of the vineyard (Gladstones, 2011; Bokulich et al., 2014). These observations opened a new branch of investigations aimed at the identification of environmental factors impacting on the composition of microbial communities and eventually on the organoleptic characteristics of the wine. Microbes can have both positive and detrimental effects on the wine fermentation process and on the organoleptic characteristics of the final product. Loureiro, Malfeito-Ferreira, and Barata proposed to group the microbes found in musts in classes highlighting their effects on fermentation: *i*- “spoilage sensu stricto” species, responsible of wine spoilage even when good practices are adopted; *ii*- “innocent species”, unable to spoil wine because controllable through the application of good manufacturing practices; *iii*- fermenting species, able to convert sugars and lactic acids, and whose presence needs to be preserved in order to achieve the fermentation (Barata et al., 2012).

The following sections will review the information obtained thanks to metagenomic approaches used to disclose the composition of microbial populations in the vineyard, in its surroundings, and in the winery, the influence of such communities on the fermentation process, and the effects of environmental factors and human intervention on microbial communities' composition.

### The vineyard

It is well known that microorganisms on and inside plant organs have an impact on the plant health, as they are involved in functions such as plant nutrition and resistance to stresses (Mendes et al., 2013). Microorganisms can promote plant growth by supplying the plants with nutrients, i.e., nitrogen, or by solubilizing substances, i.e. soluble phosphate (Lugtenberg and Kamilova, 2009). On the other hand, microbes can also have detrimental effects on plants, e.g., *Botrytis cinerea* infecting vine grapes, or saprophytic molds responsible for grape tors or mycotoxin production (e.g., *Aspergillus* spp., *Cladosporium* spp., and *Penicillium* spp.) (Barata et al., 2012). Hence, it is well known that the plant microbiota is composed of a large variety of microorganisms. However, only some of these microbes can grow in musts, and only a portion of these has a direct effect on wine production (Barata et al., 2012).

The microorganisms found in musts originate from various components of the vineyard, encompassing soil (Burns et al., 2015), air, other plants (Morrison-Whittle and Goddard, 2018), and insects (Stefanini et al., 2012; Stefanini, 2018; Table 2). The vineyard soil is one of the natural source of fungi associated with wine-related environments, with the most abundant genera being known to have an environmental origin (e.g., *Amniculicola, Doratomyces, Endocarpon*, and *Tricellulortus* (for the complete list refer to Table 2) (Morrison-Whittle and Goddard, 2018). Notably, the most abundant fungi in vineyard soil do not bear features relevant for wine production e.g., spoilage or fermentation (Barata et al., 2012). Contrarily, bacteria having various impacts on the fermentative process have been found in the vineyard soil. Among these, the most abundant are Firmicutes (encompassing fermenting, innocent and spoilage sensu-stricto species), spoiling Acidobacteria and Proteobacteria, and other bacteria having unknown effects on the fermentation such as Actinobacteria, Bacteroidetes, Chloroflexi, Gemmatimonadetes, Plactomycetes, and Verrucomicrobia (Table 2; Burns et al., 2015).

**Table 2:** Most abundant microorganisms found in vineyard and winery environments.

**Source**	**Fungi**	**Bacteria**
Vineyard-soil	**Absent/unknown effect:** *Amniculicola*^M^, *Ascobolus*^M^, *Ascodesmis*^M^, *Byssonectria*^M^, *Boudiera*^M^, *Chalara*^M^, *Chytridium*^M^, *Cordyceps*^M^, *Doratomyces*^M^, *Emericellopsis*^M^, *Endocarpon*^M^, *Flagelloscypha*^M^, *Gaertneriomyces*^M^, *Glomus*^M^, *Lamprospora*^M^, *Lasiobolidium*^M^, *Lipomyces*^M^, *Massarina*^M^, *Melastiza*^M^, *Microbotryum*^M^, *Olpidium*^M^, *Scolecobasidiella*^M^, *Sorocybe*^M^, *Spizellomyces*^M^, *Tricellulortus*^M^, *Valsonectria*^M^	**Fermenting:** Firmicutes^4^^,^^§^, **Innocent:** Firmicutes^4^^,^^§^, **Spoilage sensu-stricto:** Acidobacteria^4^, Firmicutes^4^^,^^§^, Proteobacteria^4^, **Absent/unknown effect:** Actinobacteria^4^, Bacteroidetes^4^, Chloroflexi^4^, Gemmatimonadetes^4^, Planctomycetes^4^, Verrucomicrobia^4^
Vineyard-Leaves	**Absent/unknown effect:** *Aureobasidium*^8^*, Guignardia*^8^, *Mucor*^8^, *Rhizopus*^8^, *Pandora*^8^, *Zoophthora*^8^, Dothideomycetes^7^	**Fermenting:** Firmicutes^7^^,^^§^, **Innocent:** Actinobacteria^7^, Firmicutes^7^^,^^§^, Proteobacteria^7^^,^^***^ **Spoilage sensu-stricto:** *Acidisoma*^8^, Enterobacteriaceae^8^, Firmicutes^7^^,^^§^, *Gluconoacetobacter*^8^, Proteobacteria^7^^****^, Pseudomonadaceae^8^, *Roseomonas*^8^; **Absent/unknown effect:** Streptococcaceae^8^, Moraxellaceae^8^,
Vineyard-grapes	**Fermenting:** *Hanseniaspora*^1^^,^^5^^,^^**^, Saccharomyces^1^^,^^5^; **Innocent:** *Candida*^1^^,^^5^^,^^*^, *Debaryomyces*^1^, *Hanseniaspora*^1^^,^^5^^,^^**^, Metschnikowia^1^^,^^5^^,^^9^*, Pichia*^1^^,^^***^; **Spoilage sensu-stricto:** *Botryotinia*^5^ *, Cladosporium*^5^, *Pichia*^1^^,^^***^, *Torulaspora*^1^, *Zygosaccharomyces*^1^, Saccharomycodaceae^6^; **Absent/unknown effect:** *Alternaria*^5^, *Aureobasidium*^1^^,^^5^^,^^9^*, Brettanomyces*^1^, *Cryptococcus*^5^, *Erysiphe*^5^, *Issatchenkia*^1^, *Itersonilia*^5^, *Monilinia*^5^*, Mucor*^5^*, Phoma*^5^, *Sporidiobolus*^5^, Starmerella^9^, Dothioraceae^6^, Pleosporaceae^6^, Dothideomycetes^7^	**Fermenting:** Firmicutes^7^^,^^§^, Lactobacillales^6^, **Innocent:** *Bacillales*^6^, *Bacillus*^6^, *Enterobacteriales*^6^, Firmicutes^7^^,^^§^, Proteobacteria^7^^****^, *Pseudomonadales*^6^; **Spoilage sensu-stricto:** Firmicutes^7^^,^^§^, Proteobacteria^7^^****^, Rhodospirillales^6^, **Absent/unknown effect:** *Lysinibacillus*^6^, *Sporosarcina*^6^, Pasteurellales^6^, Bacteroidales^6^, Actinobacteria^7^,
Musts	**Fermenting:** *Hanseniaspora^2^*^,^^**^, Saccharomyces^2^; **Innocent:** *Candida*^2^^,^^10^*, Hanseniaspora*^2^^,^^**^, *Lachancea*^M^, *Metschnikowia*^M^*, Pichia*^M^^,^^***^; **Spoilage sensu-stricto:** *Aspergillus*^M^*, Botryotinia*^2^*, Cladosporium*^2^*, Saccharomycodes*^M^ *, Penicillium*^2^^,^^10^, *Pichia*^M^^,^^***^; **Absent/unknown effect:** *Aureobasidium*^2^^,^^10^*, Davidiella*^2^, *Erysiphe*^M^ *, Saccharomycopsis*^M^, *Saturnispora*^M^, *Sphingomonas*^10^, *Starmerella*^10^ *Yarrowia*^M^	**Fermenting:** Lactobacillales^2^, *Oenococcus oeni*^P1^; **Spoilage sensu stricto:** Rhodospirillales^2^, **Innocent:** Bacillales^2^, Enterobacteriales^2^, Pseudomonadales^2^; **Absent/unknown effect:** Propionibacter^10^, Corynebacterium^10^
Winery surfaces (prior to harvest)	**Fermenting:** *Saccharomyces cerevisiae*^3^; **spoilage sensu-stricto:** *Aspergillus* spp.^3^; **Absent/unknown effect:** *Cryptococcus* spp.^3^*, Aureobasidium pullulans*^3^	**Innocent:** *Bacillus*^3^, *Enterobacteriaceae*^3^, *Pseudomonas*^3^; **Absent/unknown effect:** *Comamonadaceae*^3^, *Flavobacterium*^3^, *Brevundimonas*^3^,

*Microbes were classified as “fermenting,” “spoilage sensu stricto,” and “innocent” according to the (Barata et al., 2012) definition*.

1 *(Barata et al., 2012)*;

2 *(Bokulich et al., 2014)*;

3 *(Bokulich et al., 2013)*;

4 *(Burns et al., 2015)*;

5 *(Grangeteau et al., 2017)*;

6 *(Mezzasalma et al., 2017)*;

7 *(Miura et al., 2017)*;

M *(Morrison-Whittle and Goddard, 2018)*;

8 *(Pinto et al., 2014)*;

P1 *(Portillo Mdel and Mas, 2016)*;

9 *(Setati et al., 2015)*;

10 *(Stefanini et al., 2016)*;

* *considering the multi-phyletic nature of the Candida phylum further characterization at the species level is required*.

** *encompassing innocent and fermenting species*.

*** *encompassing spoilage and innocent species*.

**** *encompassing spoilage and fermenting species*.

§ *encompassing fermenting, innocent, and spoilage species. Taxa are listed at the level indicated in the referenced study*.

Another environmental source of microbes relevant for wine fermentation is the vine, in particular the bark, leaves, and obviously the grapes. An assessment carried on Portuguese vineyards (Bairrada appellation, Cantanhede) over a year revealed that leaves fungal communities were dominated by fungi belonging to the *Rhizopus, Mucor, Zoophthora*, and *Pandora* genera (Table 2; Pinto et al., 2014). While the first two genera are associated with post-harvest diseases of table grapes (Hocking et al., 2007), the two latter genera are insect-pathogenic fungi (Xu et al., 2009), and their effects on wine fermentation are unknown or absent. The presence of Ascomycota and Basidiomycota on vine leaves widely changed over time, with the most abundant being *Aureobasidium*, and *Guignardia* (a phytopathogen) (Pinto et al., 2014). The fermenting species *Saccharomyces, Hanseniaspora*, and *Metschnikowia* have also been identified on vine leaves, though at low frequencies (Pinto et al., 2014). The most abundant bacterial families on vine leaves are Streptococcaceae, Enterobacteriaceae, Pseudomonadaceae, and Moraxellaceae (Pinto et al., 2014). Only a few Lactic Acid Bacteria (LAB), responsible for malolactic fermentation, have been identified at low frequencies on vine leaves (*Lactobacillaceae* family; Table 2; Pinto et al., 2014). Furthermore, Acetic Acid Bacteria (AAB), known to spoil wine fermentations (Drysdale and Fleet, 1988), such as the genera *Acidisoma, Gluconoacetobacter*, and *Roseomonas*, are predominantly present on leaves (Pinto et al., 2014). Noticeably, the fungal biodiversity on vine leaves show a tendency to decrease over time (Pinto et al., 2014). This reduction of biodiversity can be ascribed to various factors: repeated chemical treatments, routinely used in conventional viticulture (see section Anthropogenic Factors Influencing Microbial Populations; Pinto et al., 2014); seasonal/climatic changes (see section Environmental Factors Influencing Microbial Populations); the emergence of fruit, a potentially more suitable habitat than leaves for molds and fungi because rich in sugars (Bokulich et al., 2014; Grangeteau et al., 2017).

In spring vine fertilized flowers start to develop a seed and a grape berry to protect it. Grape growth and maturation occur in the following months, with a duration changing according to the climate (Perrot et al., 2015). While growing and ripening, grapes are exposed to microbes originating from the surrounding environment, and the microbial communities on grape skins are subjected to dynamic changes due to environmental factors and anthropogenic interventions. Being the only ingredient for wine production, harvested grapes are the major source of microbes contributing and affecting the fermentation. *Mucor* and *Aureobasidium* have been identified among the most abundant fungal genera in grapes (Table 2; Grangeteau et al., 2017). In addition, grape fungal populations also show high levels of fungal genera known to variously affect the fermentation process: fermenting genera (*Saccharomyces*), “innocent” genera (e.g., *Debaryomyces*), spoilage *sensu stricto* genera (*Brettanomyces, Cladosporium*, Saccharomycodaceae), genera encompassing spoilage and fermenting yeasts (*Torulaspora*, and *Zygosaccharomyces*), and also genera whose impact on fermentation is unknown (e.g., *Alternaria*)(full list of genera in Table 2; Barata et al., 2012; Grangeteau et al., 2017; Mezzasalma et al., 2017). Acetic acid bacteria have been found at low frequencies in grape samples (Portillo Mdel et al., 2016), but still potentially affecting the outcome of fermentation.

The composition of fungal populations in grapes has been found to be associated with the geographical location of the vineyard, thus further supporting the concept of microbial terroir (Pinto et al., 2014; Bokulich et al., 2016; Miura et al., 2017; Morrison-Whittle and Goddard, 2018). The geographical diversification of fungi has been observed when comparing the complete population structure, and none of the identified fungal species had a geographic specificity, being either more abundant or present in only one of the compared locations (Bokulich et al., 2016; Miura et al., 2017). The geographical diversification observed for grape fungal populations has been observed also for grape bacterial populations (Portillo Mdel et al., 2016; Mezzasalma et al., 2017). However, some bacteria have been constantly found at high frequencies in grapes: Lactobacillales (fermenting), Bacillales, Enterobacteriales, and Pseudomonadales (innocent), Actinomycetales and Rhodospirillales (Portillo Mdel et al., 2016; Mezzasalma et al., 2017). Bacillales have been identified at high frequencies in all the grape samples analyzed in both the Mezzasalma et al. (2017) and Portillo Mdel et al. (2016) studies, encompassing various vine varietals, geographical locations, and vineyard orientations (Portillo Mdel et al., 2016; Mezzasalma et al., 2017). Contrarily, the presence and abundance of other bacterial genera and families have been found to be associated with either the vineyard orientation (South, East, or flat) or the vine varietal (further details in section Environmental Factors Influencing Microbial Populations; Portillo Mdel et al., 2016).

### The winery and the fermentation process

**Figure 2:**
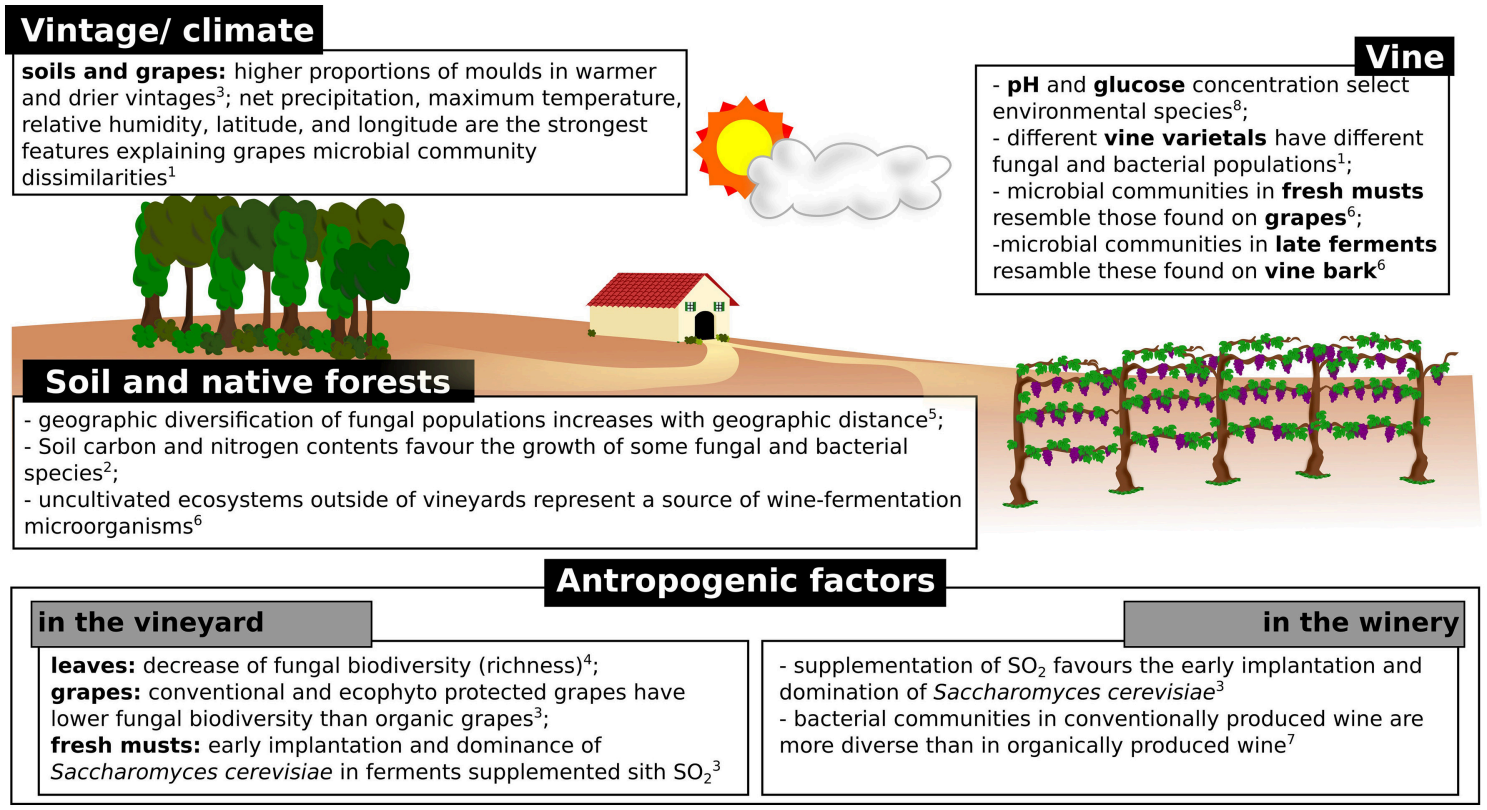
Schematic representation of the factors known to influence the composition of microbial populations involved in wine fermentations. ^1^(Bokulich et al., 2014); ^2^(Burns et al., 2015); ^3^(Grangeteau et al., 2017); ^4^(Pinto et al., 2014); ^5^(Miura et al., 2017); ^6^(Morrison-Whittle and Goddard, 2018); ^7^(Piao et al., 2015); ^8^(Stefanini et al., 2016).

The conversion of must into wine is a dynamic process involving numerous transformations carried out by a complex succession of yeast and bacterial species. The process is achieved in two steps: alcoholic fermentation, generally carried out by yeasts, followed by malolactic fermentation, conducted by bacteria (Cappello et al., 2017). It is well known that alcoholic fermentation is carried out by a few yeast species, which eventually overcome the microbial population present in must because of the sensitivity to high ethanol concentrations and temperature of most microorganisms (Goddard, 2008). In general, the increase of temperature and ethanol concentration during fermentation induces a decrease in population complexity (e.g., richness), while the size of the population continues to increase, due to the overcome of the resistant species (Stefanini et al., 2016). In a recent study, Morrison-Whittle and Goddard highlighted the high similarity of vineyard and must fungal populations, with approximately the 40% of the fungal communities present in musts and during fermentation being also present in vineyard samples (soil, vine bark, and ripe fruit) (Morrison-Whittle and Goddard, 2018). The clear majority of fungi found in musts are also found in grapes, such as the genera *Aureobasidium, Botryotinia, Candida, Cladosporium, Columnosphaeria, Davidiella, Hanseniaspora*, and *Saccharomyces* (Bokulich et al., 2014; Morrison-Whittle and Goddard, 2018; Table 2). In addition, approximately the 30% of species present during fermentation were also present in samples (soil and fruit) collected from native conservation reserves located nearby the studied vineyards (Figure 2), thus highlighting the relevance of preserving uncultivated areas nearby the vineyards to safeguard the maintenance of fungal biodiversity in fermentations (Morrison-Whittle and Goddard, 2018).

Other fungal genera can be found in must samples, but rarely persist during the fermentation, both because of the environmental changes and thanks to the adoption of techniques aimed at the control of spoiling species. Among the species found in musts and rarely persisting, the most abundant are usually *Pichia* (encompassing both “innocent” and “spoilage *sensu stricto*” species), *Aspergillus* (considered a spoiling fungus as producing ochratoxins), *Saturnispora, Saccharomycopsis, Saccharomycodes* (spoilage genus), *Yarrowia, Erysiphe*, and *Metschnikowia* (the latter is an “innocent” and “fermenting” genus; Morrison-Whittle and Goddard, 2018; Table 2). As fungal populations, bacterial populations in the vineyard and must have been shown to be highly similar (Bokulich et al., 2014; Portillo Mdel and Mas, 2016). Bacterial populations in the musts are dominated by Bacillales, Enterobacteriales, Lactobacillales, Pseudomonadales, and Rhodospirillales, with a higher proportion of LAB than what observed on vine leaves (Bokulich et al., 2014; Portillo Mdel and Mas, 2016; Table 2).

Noticeably, the clear geographical diversification of microbial populations observed in musts weakens during fermentation (Morrison-Whittle and Goddard, 2018), probably because of a collapse of microbial diversity as *Saccharomyces* yeasts displace other species (Goddard, 2008). Nevertheless, as the fermentation process proceeds, must populations have been shown to increasingly resemble those found on vine barks, possibly due to the high frequency of *Saccharomyces* spp. yeasts found in both fermentation and bark samples (Morrison-Whittle and Goddard, 2018). *Saccharomyces cerevisiae* is almost always the species dominating the fermentation, but other yeast species (*Candida* spp. and *Hanseniaspora* spp.) have been shown to be present at high frequencies, especially during the early phases of the process (Portillo Mdel and Mas, 2016; Stefanini et al., 2016). In some occasions *Candida* spp and *Hanseniaspora* spp have also been shown to be able to dominate the fermentation (David et al., 2014; Stefanini et al., 2016).

A great step forward in our understanding of bacterial populations composition during alcoholic fermentation was achieved thanks to the application of metagenomic approaches. In fact, studies carried out before the advent of metagenomics suggested some bacterial species could not persist during alcoholic fermentation due to their sensitivity to alcohol: according to culture-based studies, the abundance of AAB was considered to decrease from 10^6^-10^7^ colony forming units (CFU)/ml in must to 10^2^-10^3^ CFU/ml at the end of alcoholic fermentation (Du Toit and Pretorius, 2002). Contrarily, the use of culture-independent methods reported that the high abundances of AAB, in particular of *Gluconobacter* (Acteobacteraceae), remained elevated throughout fermentation (Andorrà et al., 2010; Bokulich et al., 2012; Portillo Mdel and Mas, 2016). Nevertheless, the abundances of AAB detected by means of metagenomic approaches were shown to decrease with fermentation, possibly also because of the increase of LAB abundances (Portillo Mdel and Mas, 2016). In general, the inoculation of the yeast *S. cerevisiae* strains, a practice currently used to support and control the fermentation, has been shown to largely impact on the composition of bacterial populations, and to reduce the biodiversity, inducing a reduction of acetic acid bacteria (Bokulich et al., 2012).

As previously described for the vineyard, also the winery environment is a source of microorganisms involved in must fermentation (Bokulich et al., 2013; Belda et al., 2017). Under normal cleaning conditions, large populations of fungi and bacteria are found on winery surfaces prior to harvest (Table 2) (Bokulich et al., 2013). Such persistent microorganisms encompass fermenting, spoiling and innocent fungi and bacteria: *Pseudomonas, Enterobacteriaceae, Bacillus, S. cerevisiae, Cryptococcus* spp., *Aureobasidium pullulans*, and *Aspergillus* spp. (Bokulich et al., 2013; Table 2). Hence, these microorganisms will potentially contribute to the fermentation process of the following vintage.

### Environmental factors influencing microbial populations

Among the known environmental factors known to influence the microbial populations found in various vineyard specimens, the vintage is probably the most relevant (Figure 2). The number and type of taxa identified in grape samples are associated with vintage characteristics, including factors such as temperature and rainfall (Bokulich et al., 2014; Grangeteau et al., 2017). Grangeteau and collaborators showed that the total number of fungal species and the proportion of molds were greater in warmer and drier vintages compared to cold vintages with heavy precipitations (Grangeteau et al., 2017). In addition, the *Botryotinia, Cladosporium*, and *Phoma* genera were found only in warm and dry vintages, while the *Monilinia* genus was found in vintages with lower temperatures and greater precipitations (Grangeteau et al., 2017). Similarly, Bokulich and collaborators showed the vintage effect on microbial populations present in must samples (Bokulich et al., 2014; Figure 2), with maximum temperature and relative humidity being among the strongest features explaining microbial community dissimilarities across grape microbial community patterns (Bokulich et al., 2014).

Other factors shaping the composition of wine fermentation-related microbial populations are the physical characteristics of the vineyard. Burns and collaborators showed that high abundances of Alphaproteobacteria and Actinobacteria families were found in vineyard soils having high contents of carbon or nitrogen (Burns et al., 2015). Contrarily, Sphingomonadaceae, Comamonadaceae, Pseudomonadaceae, Xanthomonadaceae, Micrococcaceae, Nocardiaceae, Flavobacteriaceae, Bacillaceae, and Paenibacillaceae were more abundant in soils showing low amounts of carbon or nitrogen sources (Burns et al., 2015). Unfortunately, the analyzed vineyards, located in the Napa Valley (California), showed several characteristics correlated with each other (i.e., elevation was positively correlated with latitude, slope, and average annual precipitation), hence probably preventing the identification of further associations (Burns et al., 2015). However, the topological characteristics of the vineyard have been shown to greatly influence the composition of wine-related microbial populations (Portillo Mdel et al., 2016). In fact, *Pseudomonas* (an innocent genus), Haemophilus, Oxalobacteraceae, *Sphingomonas*, have been shown to be constantly present in grape samples from vineyards exposed to East, while *Staphylococcus* (innocent genus), *Streptococcus*, Micrococcaceae, Enhydrobacter, and Aeromonadaceae have been shown to be typical of flat vineyards (Portillo Mdel et al., 2016).

Notably, it has been shown that the vine cultivar influences the composition of fungal and bacterial populations (Bokulich et al., 2014). Bokulich and collaborators showed that *Capnodiales, Protobacteria*, and *Penicillium* were more abundant in Chardonnay grapes, Cabernet Sauvignon grapes were enriched in β-*Proteobacteria, Bacteroidetes, Clostridia, Dothideomycetes, Agaricomycetes, Tremellomycetes, Microbotryomycetes*, and *Saccharomycetaceae*; and *Firmicutes, Gluconobacter, Eurotiomycetes* (*Aspergillus*), *Leotiomycetes*, and *Saccharomycetes* were more abundant in Zinfandel (Bokulich et al., 2014).

In addition, must chemical-physical factors have been shown to play a relevant role in selecting microbial populations. For example, acidic musts (low pH) show high amounts of the environmental species *Pichia membranifaciens*, whereas *Wickerhamomyces anomalus, Pichia bialowiezense, Guehomyces* spp., *Cladosporium* spp., *Torulaspora delbrueckii*, and *Nakazawaea holstii* showed a preference for an environment characterized by a low ethanol concentration, high glucose concentration, and mildly acidic pH, as is usually the case for must in the early stages of fermentation (Stefanini et al., 2016). However, the composition of microbial populations is highly dynamic during the conversion of must into wine, and several studies have been done to dissect the dynamics of microbial populations. In general, the richness of both bacterial and fungal populations decreases during the process (Pinto et al., 2015). The fungal population, which is dominated by environmental species in musts, shows an initial growth of non-*Saccharomyces* (i.e., *Hanseniaspora, Metschnikowia, Pichia*, and *Torulaspora*). Later, the number of species is reduced, and a few yeast species are abundant in spontaneous fermentations: *S. cerevisiae, Candida zemplinina, Hanseniaspora* spp., *Metschnikowia* spp., and *Lachancea* spp. (Pinto et al., 2015; Stefanini et al., 2016).

### Anthropogenic factors influencing microbial populations

Aiming to the optimization of the product, winemakers intervene in several stages of the process, from the vineyard up to the winery. The most common human interventions encompass the decision of using protective treatments in the vineyard, inoculating musts with either selected microorganisms or enriched environmental populations (*pied de cuve*), or adding chemicals to the must to eradicate spoiling microorganisms. Several different farming approaches are nowadays adopted in the vineyard, among which the most extreme are the conventional, based on the application of chemical fungicides and biofertilizers, and the biodynamic and organic approaches, avoiding the use of pesticides and herbicides. Some efforts have been made to evaluate the effects of these approaches on microbial communities associated with wine production. The fungal richness in grapes was found to be higher in conventional and ecophyto (same compounds used in conventional protection, at a lower dosage) than in organic (treated with only pyrethrin, copper, and sulfur) vineyards (Grangeteau et al., 2017). Basidiomycota (especially *Cryptococcus*) were mainly found in organic vineyards, as well as *Fusarium* and *Mucor*, whereas the fermenting genera *Saccharomyces, Metschnikowia*, and *Hanseniaspora* are mainly associated with the conventional method (Grangeteau et al., 2017). The effects of the farming approaches were also observed in microbial populations found in the must. In fact, the fungal biodiversity was found to be higher in musts from biodynamic vineyards (treated with sulfur, copper oxide, organic fungicides) than in conventional (chemical fungicides and biofertilizers are applied) and integrated (application of biofertilizers, mycorrhizae, combination of systemic and surface protectants for pest control) vineyards (Bagheri et al., 2015).

Conventional farming approaches make use of repetitive and various chemical treatments in the vineyard, which have been shown to influence both fungal and bacterial communities present on vine leaves (Pinto et al., 2014) and grapes (Setati et al., 2015). Chemical treatments affect the microbial biodiversity, especially reducing the relative abundances of *Aureobasidium* spp., *Cryptovalsa, Bulleromyces, Diaporthe*, and increasing the relative abundances of *Alternaria, Claviceps, Guignardia, Lewia, Puccinia, Sporormiella, Stemphylium*, and *Ustilago* on leaves (Pinto et al., 2014). When different combinations of chemicals were sequentially applied in the vineyard, each treatment was shown to affect the whole fungal community (Pinto et al., 2014). After treatments with chemicals encompassing the active element sulfur, a noticeable reduction was observed for the abundances on vine leaves of the genera *Aureobasidium, Rhodotorula*, and *Candida* (Pinto et al., 2014). In addition, the abundance of *Aureobasidium* was also affected by treatments supplemented with folpet, an agricultural fungicide used for the control of downy and powdery mildew and gray mold infections (Pinto et al., 2014). Concerning bacteria, chemical treatments have been shown to decrease the relative abundances of Enterobacteriaceae, Pseudomonadaceae, Comamonadaceae and Xanthomonadaceae families (Pinto et al., 2014; Figure 2).

Aiming to the control of spoilage microorganisms, winemakers have adopted in the winery a series of protocols including the control of temperature, the inoculation of *S. cerevisiae* strains, and the supplementation of musts with chemicals (i.e., SO_2_). The inoculation of *S. cerevisiae*, a technique adopted since the mid-late nineteenth century (Muller-Thurgau, 1896), is aimed at exploiting the vigorous fermentative capacity of this species to obtain a very efficient ethanol production and impose the inoculated strain over the rest of the microbiota, potentially able to spoil the wine (Piskur et al., 2006). The inoculation of *S. cerevisiae* reduces the biodiversity of microbial populations, and in particular of acetic acid bacteria, possibly by increasing the fermentation rate and the must temperature consequently (Bokulich et al., 2012). Currently, winemakers are interested in using non-*Saccharomyces* yeasts during alcoholic fermentation to increase wine complexity and differentiation (Lleixà et al., 2016). To meet this requirement, companies have started to study and commercialize *Torulaspora delbrueckii* and *M. pulcherrima* (Jolly et al., 2014). In addition, researchers have started to investigate the possibility to exploit one of the non-*Saccharomyces* genera most abundant in grape must, *Hanseniaspora*, and *H. vinae* has so far shown the most promising potential as fermentation starter (Lleixà et al., 2016). Interestingly, the inoculated *H. vinae* strains were shown to persist at high frequencies in musts only during the initial days of fermentation, and, despite being overturned by natural *S. cerevisiae* strains, were able to modify the organoleptic properties of wine (further details in section Effects of Microbial Populations on the Quality of Wine Fermentation; Lleixà et al., 2016).

Grangeteau and collaborators showed that the human intervention during the fermentation process (on musts) can modify the composition of microbial populations with a reduced impact than the human activities in the vineyard (Grangeteau et al., 2017). Indeed, the type of protection applied in the vineyard (conventional, ecophyto or organic) was shown to have the major effect on the dynamics of fungal populations during the fermentation (Grangeteau et al., 2017). However, also the supplementation of musts with SO_2_ had an effect, favoring the early implantation and domination of the genus *Saccharomyces* (Grangeteau et al., 2017). Similarly, bacterial communities were shown to be affected by the supplementation of SO_2_ (Bokulich et al., 2015; Piao et al., 2015). Bokulich and collaborators showed a dose-dependent effect of SO_2_, with 25 mg/l SO_2_ being the minimal concentration required to stabilize the bacterial population, also resulting in the control of *Gluconobacter* and LAB (Bokulich et al., 2015). However, the same study also revealed that the inoculation of *S. cerevisiae* had the same effect of SO_2_ on bacterial populations and that the effect was not additive with the supplementation of SO_2_ (Bokulich et al., 2015). A similar result was reported by Piao and collaborators, revealing higher abundances of the spoiling *Gluconobacter oxydans* and, in a minor extent, *Acetobacter*, in organic fermentations (not supplemented with SO_2_), compared to conventional fermentations (supplemented with 55.8 mg/L SO_2_; Piao et al., 2015).

### Effects of microbial populations on the quality of wine fermentation

As described in previous sections, a wealth of studies based on metagenomic approaches have investigated microbial populations associated with wine production, not only to describe them, but also to identify factors affecting their compositions. Contrarily, only a few studies have explored the associations between microbial communities and wine organoleptic characteristics (Bokulich et al., 2016; Lleixà et al., 2016; Stefanini et al., 2017b). It is worth mentioning that most of current studies on the associations between microbial communities and organoleptically relevant compounds were aimed at identifying correlations, without claiming causation. In other words, the identification of positive or negative correlations does not mean that the microbe produces (positive correlation) or is killed/controlled by (negative correlation) the compound. Rather, correlations could be potential markers to predict wine metabolite composition (Bokulich et al., 2016). Further studies should be done to assess the potential role of microorganisms in flavor production (Bokulich et al., 2016).

The geographical differentiation observed for microbial populations was also observed for wine metabolites (Knight et al., 2015; Bokulich et al., 2016). This observation encouraged Bokulich and collaborators to search for correlations between microbial (fungal and bacterial) genera abundances and metabolite amounts (Bokulich et al., 2016). Noticeably, associations were identified between Leuconostocaceae (with *O. oeni* as the best sequence hit) and a metabolite tentatively assigned as methyl benzoate, phenylacetate, or p-anisaldehyde, between *Hanseniaspora uvarum* and a metabolite tentatively identified as acetophenone, phenylacetaldehyde, or 3-methyl benzaldehyde, and between *Pichia guilliermondii* and a two metabolites identified as octanoic acid and C_6_H_10_O_2_ (either acid, ester, or lactone) (Bokulich et al., 2016). Noticeably, several of the compounds identified as being associated to microbial species are known to have scents lending wine either pleasant or unpleasant characteristic, e.g., methyl benzoate has pungent, heavy, floral odor with fruity undertones; p-anisaldehyde has an intensely sweet floral odor; phenylacetaldehyde has a rose-like scent; octanoic acid has an unpleasant odor [information obtained from PubChem (Kim et al., 2016) and “the good scent company” website, http://www.thegoodscentscompany.com/]. Other correlations have been identified among fungal genera and volatile compounds in withering *V. vinifera* L. cv. Corvina grapes and musts of Amarone, a dry wine produced exclusively in the Italian region of Valpolicella (Verona) (Stefanini et al., 2017b). The fungal genus *Phoma*, found at high frequencies in withering Corvina grapes, showed a positive correlation with (3E)-3-hexenoic acid. The *Diplodia* genus, highly abundant in musts, was found to be positively correlated with 1-pentanol (amyl alcohol, having a balsamic, fusel, oil, sweet, vanilla flavor) and 2,6-dimethoxy phenol (syringol, having a bacon, balsamic, phenol, powdery, smoke, woody flavor). Contrarily, other genera highly abundant in musts showed negative correlations with volatile compounds known to have a relevant impact on wine aroma. The genus *Candida* showed a negative correlation with *p*-formilphenol, having an almond, balsam, sweet, woody flavor, and dichloromethane, having a sweet smell. The *Cytospora* genus showed a negative correlation with paraldehyde (aromatic and sweet smell), and tetradecane (alkane, mild, waxy smell). The genus *Metschnikowia* was found to have negative correlations with (3E)-3-hexenoic acid (acid, cheesy, fruity, grass, sweaty flavor), isoamyl acetate (banana and pear), dibutyl phthalate (faint smell), paraldehyde (sweet and aromatic smell), p-formaldehyde (almond, balsam, sweet, woody smell), triethylene glycol (odorless, but potentially acting as disinfectant), and dichloromethane (sweet smell). Both *Cytospora* and *Metschnikowia* showed negative correlations with caprylic acid (cheesy, rancid smell) and octadecane (alkane smell).

As previously stated (section Anthropogenic Factors Influencing Microbial Populations), not-*Saccharomyces* strains are being studied as potential starters (aka strains inoculated in musts to promote alcoholic fermentation) to increase wine complexity and differentiation. The inoculation of *Hanseniaspora vinae* was shown to modify the organoleptic characteristics of wine, despite the inoculated strain was rapidly replaced by natural *S. cerevisiae* strains present in the must (Lleixà et al., 2016). In particular, the amounts of N-acetyl tyamine and 1H-indole-3-ethanol acetate ester, usually not found in wine fermentations, were found only in musts inoculated with *H. vinae*, and phenethyl acetate, conferring floral, fruity and honey-like aromas to wine, was 50 times more abundant in wines fermented with *H. vinae* (Lleixà et al., 2016). Noticeably, Lleixà and collaborators also reported that wine-tasters selected and easily distinguished wines fermented with *H. vinae*, indicating that the early presence of this species can greatly modify the characteristics of the wine (Lleixà et al., 2016).

## Future challenges for metagenomic approaches: the sub-species level

Amplicon-based approaches allow us to obtain a general picture of the microbiota but have a taxonomic resolution that, in the best situations, assigns individuals at the species level (Stefanini et al., 2016). Although this might be sufficient to describe and compare populations at the large scale, in some situations a higher resolution is necessary. For instance, *S. cerevisiae* isolated from different geographical locations have shown different genetic and phenotypic characteristics, thus suggesting the existence of geographically-specific lineages of this yeast (Yarza et al., 2014). Hence, the disclosure of microbial populations at the strain level is of great interest to better understand the distribution and diffusion of microorganisms from the vineyard to the winery and among vineyards.

Aiming to identifying different strains of a given species, a few culture-independent procedures have been developed. Among these procedures, MetaMLST (Zolfo et al., 2017) and *S*ID (Stefanini et al., 2017a) are based on approaches used to identify isolates by means of genetic markers, MLST (multilocus sequences typing) and microsatellites sequencing, respectively. MetaMLST allows the identification of strains by comparing whole-genome metagenomic sequences with databases of species-specific loci (Zhang et al., 2016). Contrarily, *S*ID is based on the use of microsatellites, non-coding DNA sequences composed by small repeated units (2–6 bp) which are repeated a variable number of times in different individuals (Legras et al., 2005). Hence, *S*ID identifies the combination of microsatellite profiles of strains from a reference dataset most likely composing the microsatellite profile obtained on a complex sample (e.g., microbial DNA extracted from must, grapes; Stefanini et al., 2017a). MetaMLST and *S*ID enable the identification of different strains according to the similarity of the sample profile to the profiles present in reference databases. The use of MetaMLST to wine fermentation is currently limited due to the availability of MLST databases enriched in bacterial and fungal species of clinical interest (Zolfo et al., 2017), thus making this approach not suitable for wine-related samples. On the contrary, microsatellite sequencing has been widely used to type microorganisms in fermentation, but most of such studies were limited to the *S. cerevisiae* species (Legras et al., 2005, 2007; Ezov et al., 2006; Richards et al., 2009).

Recently, another tool has been proposed by the Segata group, StrainPhlAn (Truong et al., 2017). StrainPhlAn is based on reconstructing consensus sequence variants within species-specific marker genes identified for MetaPhlAn2 and building a phylogenetic tree on the consensus sequences to identify different strains (Truong et al., 2015). The species-specific markers (~1 million markers from >7,500 species) (Truong et al., 2017) used in MetaPhlAn analyses have been identified by comparing the genomes available from the Integrated Microbial Genomes system, encompassing publicly available bacterial, archaeal, eukaryotic, and phage genomes, as well as engineered, environmental and host-associated microbiome samples (Truong et al., 2015). Hence, since it is not biased toward clinically-relevant microbes, this approach holds a great potential in supporting the identification of microbial strains present in wine-related metagenomes.

## Conclusions

Metagenomic approaches are largely contributing to the dissection of the so-called “microbial terroir,” microbial communities typical of the geographical area of wine production. Thanks to these approaches, the rapid and exhaustive characterization of microbial populations present in various specimens associated with vineyards, wineries, and fermentation is nowadays possible. In addition to evaluating the existence of a microbial terroir, new studies allowed the identification of several environmental and human-related factors influencing the composition of microbial populations, and hence potentially affecting their fermentative performances. And yet, despite the great contribution made by these studies, the microbial spreading and persistence from the vineyard to the winery are still far from being completely dissected. Further studies, exploring a wider variance of vine varietals, comparing different procedures (adopted in the vineyard and in the winery) and different environments, will increase our knowledge of this complicated process. Probably one of the most complex achievement is the separation of topological variables characterizing the vineyard and environmental variables characterizing a “vintage.” A proper comparison of microbial populations in environments varying by only one or few variables will help in this goal. In addition, the observation of clear geographic diversification of fungal populations, and weaker diversification of bacterial communities may indicate the need for understanding the role of vectors in moving microbes across areas. Indeed, while bacterial and fungal spores can blow in the wind and be transported among distant geographic locations, not-airborne yeasts require animals to be vectored among distant (by birds) or close (by insects) locations (Francesca et al., 2012; Stefanini et al., 2012). A complete survey of the microbiota of these vectors will help in completely understanding the fluxes of microorganisms relevant for wine fermentation. The complete understanding of all the factors influencing the composition of microbial populations and their passage from the vineyard to fermenting musts will help winemakers by disclosing the association between variables and outcomes, thus allowing the adoption of the most appropriate techniques according to environmental changes.

## Author contributions

IS wrote the first draft of the review. DC proofed the drafts. IS and DC finalized the review.

### Conflict of interest statement

The authors declare that the research was conducted in the absence of any commercial or financial relationships that could be construed as a potential conflict of interest.
